# Plasma Parameters around a Chain-Like Structure of Dust Particles in an External Electric Field

**DOI:** 10.3390/molecules26133846

**Published:** 2021-06-24

**Authors:** Mikhail Salnikov, Alexander Fedoseev, Gennadiy Sukhinin

**Affiliations:** Institute of Thermophysics SB RAS, 630090 Novosibirsk, Russia; miror1994@mail.ru (M.S.); sukhinin@itp.nsc.ru (G.S.)

**Keywords:** complex plasma, dust particles interaction, chain-like structure, interparticle distance

## Abstract

The formation of a 1D chain-like structure of dust particles in a low-temperature argon plasma was studied. A new numerical model for calculation of the self-consistent spatial distribution of plasma parameters around a chain of dust particles was presented. The model described the motion of positively charged ions in the electric potential of several negatively charged dust particles, taking into account the action of an external electric field. The main advantage of the model was that the charges of the dust particles and the interparticle distances were determined self-consistently. As a result of numerical simulations, the dependencies of the spatial distributions of the plasma parameters (the densities of electrons and ions and the self-consistent electric potential) near the dust particles chain on the strength of the external electric field, an external force acted on the last particle, and the mean free path of the ions was determined. The obtained results made it possible to describe the process of the formation of chain-like structures of dust particles in discharge plasma.

## 1. Introduction

Dusty plasma is a complex medium observed experimentally by Langmuir at the beginning of the twentieth century [1]. This medium is characterized by the interaction of ordinary plasma with the solid particles placed in it [2]. Dusty plasma is widespread in space [3,4,5,6], laboratory environment, and industrial devices [7,8]. The spontaneous appearance of the dust particles in industrial devices could lead to negative consequences—for example, the substrates contamination in chemical vapor deposition [9]—which is why it is necessary to study it. One of the intriguing phenomena that occur in dusty plasma is the self-organization of dust particles into structures resistant to external influences, i.e., dust crystals [10,11,12,13,14]. Along with the formation of a 2D monolayer of the dust particles with a hexagonal crystal lattice, numerous studies have been carried out about the alignment of dust particles into 1D chains [15,16,17,18,19,20,21,22,23,24].

To date, many experimental works have been published whose purpose was to obtain and study dust particle chains in a laboratory environment [25,26,27]. In these works, chain characteristics and their dependence on the discharge parameters were investigated. However, there are still questions that are difficult to answer by performing only the experiments. The main questions are: what the reasons are for establishing certain distances between the particles in a chain, dependence of the interparticle distance on the discharge parameters, why in the AC discharge mode the chains are less stable than in the DC mode, and why larger particles form chains more easily [28]. Due to the technical limitations of the experimental equipment, more detailed studies of the plasma dynamics around dust particles are often carried out using analytical methods or numerical models. Of the large number of theoretical models, it is especially important to highlight the following.

An analytical model was presented in reference [29], where the potential energy of two dust particles interacting in a plasma flow was investigated. Using this model, in reference [30], taking into account the gravity force, the regions of the parameters for which the structure of the dust chain was stable were calculated. It was also shown that the structure of the dust chain becomes highly unstable when dust particles have the same charge. Though this model calculates the boundaries of parameters where the dust particles can still form stable chains, it does not take into account the final sizes of dust particles, the self-consistent charging of dust particles, and plasma dynamics, which makes it impossible to determine self-consistently the interparticle distances.

In references [31,32], a model was presented in which a thorough study of the dust particles interaction was performed on the basis of the potential that includes the analytical form of the dielectric constant. In this model, the motion equations of an infinite chain of dust particles in subsonic and supersonic plasma flows were considered. This study determined the parameter intervals in which the dust particle chain was stable. The model [31] potentially made it possible to take into account the collisions of ions and electrons with neutral atoms and to study the dynamics of a finite chain of dust particles. However, within the framework of this model, it was extremely difficult to self-consistently calculate the charge of dust particles and to take into account their finite size. In addition, it is worth noting that the regions of stability presented in the models from references [30,31] did not fully coincide.

The main numerical model used to study clusters and chains of dust particles is the PIC method [33]. The latest modification of PIC is DiP3D, presented in references [34,35,36,37]. This three-dimensional model is used to study systems in which the dust particle structure is flowed around by a plasma flow. It is based on the calculation of the trajectories of ions and electrons in a self-consistent spatial distribution of the potential of dust particles screened by a space charge. DiP3D allows to calculate self-consistently the dust particle charging, elastic and resonant collisions of ions and electrons with neutral atoms, and even investigates the motion of ions and electrons in a magnetic field [36]. Using this model for a collisionless plasma, the characteristics of the wake behind an isolated dust particle were determined depending on the ion flow velocity [33]. The dependence of the dust particle charges and the interparticle distances in a chain were also calculated for supersonic and subsonic flows. The main disadvantages of this method are: its high calculation cost, primarily associated with the slow computation of the Poisson equation [34]; the computational accuracy is reduced for certain intervals of parameters, which leads to instabilities for certain Mach numbers [37]; difficulties in constructing a sufficiently detailed mesh in the computational domain [34]; and the self-consistent distance between the dust particles of the chain is calculated as the integral of the instantaneous distribution of the space charge density over a limited part of the space occupied by the wake [35].

Based on the above, at the moment, the question of the self-consistent organization of the dust particle chains remains not fully resolved. What will be the dependence of the charge of dust particles in the chain and the interparticle distance between them on the plasma parameters, and would such a chain will be stable? In references [38,39], a model was presented where the dependence of the spatial distributions of plasma around an isolated dust particle on various parameters of the particle itself, the surrounding plasma, and the external electric field was calculated. It was shown in reference [28] that the approximation of the dipole moment obtained in reference [38] can be used to describe with a good approximation the range of parameters in which dust particles self-organize into a chain.

This paper presents a new model, an extension of the previous model [38,39], which includes the calculation of the spatial distributions of plasma around the chain of dust particles, the self-consistent charging of several dust particles, and the calculation of the self-consistent interparticle distance in a finite chain of dust particles.

## 2. Results and Discussion

In this paper, the self-organization of a one-dimensional dust particle chain placed in a low-temperature argon plasma was investigated. The maximum possible number of dust particles that could be considered in the presented model is limited only by the hardware computational capability. However, in this paper, a chain consisting of three dust particles (*N* = 3) was investigated. The electron–ion temperatures ratio was set as *τ* = *T_e_*/*T_i_* = 100 (the ion temperature is *T_i_* = 0.023 eV). The value range of the mean free path of resonant charge exchange collisions was *l_i_* = 5–20 *λ_i_*, and for the dimensionless external electric field strength, it was *E* = 0–0.125. With the specified parameters and the ion Debye length *λ_i_* = 100 µm, the considered range of gas pressures was *p_g_* = 1–5 Pa, and the external electric field strength was E = 0–0.3 V/cm. It is also worth noting that dimensionless dust particle charge value *Q* = 1 corresponded to *Z_d_* ≈ 1600 *e*. For convenience, all further variables will be presented in the dimensionless form.

**Figure 1:**
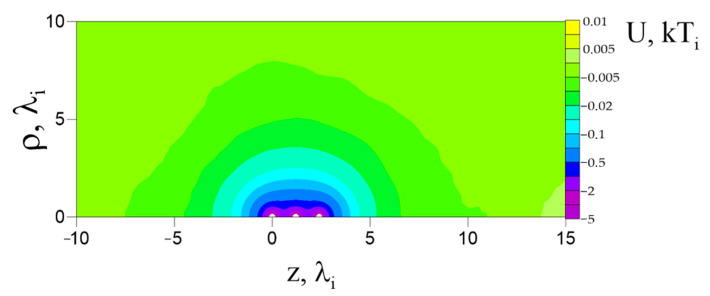
Self-consistent spatial distribution of the potential *U*(*ρ*, *z*) in the vicinity of a dust particle chain. *F_ext_* = −0.2, *l_i_* = 5, *E* = 0.

Block 1 calculated the space charge *n*(*ρ*, *z*) and plasma potential *U*(*ρ*, *z*) spatial distributions around a given dust particle chain. These distributions determined the forces with which the surrounding plasma acted on the dust particles. In Figure 1, a self-consistent plasma potential spatial distribution *U*(*ρ*,*z*) calculated in the vicinity of a chain of three dust particles is presented. This distribution is calculated for the confining force *F_ext_* = −0.2 and ion mean free path *l_i_* = 5 in the absence of an external electric field *E* = 0. This distribution is an example of electrostatic screening of a dust particle chain by the surrounding plasma. The screening process for a dust particle chain is identical to the screening process for an isolated dust particle [39]. When solid dust particles are injected into plasma, electrons and ions collide with them, and the dust particles acquire a negative electric charge due to the greater mobility of electrons. Positively charged ions begin to orbit around the negatively charged dust particles, forming an ion cloud, which screens the negative charge of the particles (ion shielding).

**Figure 2:**
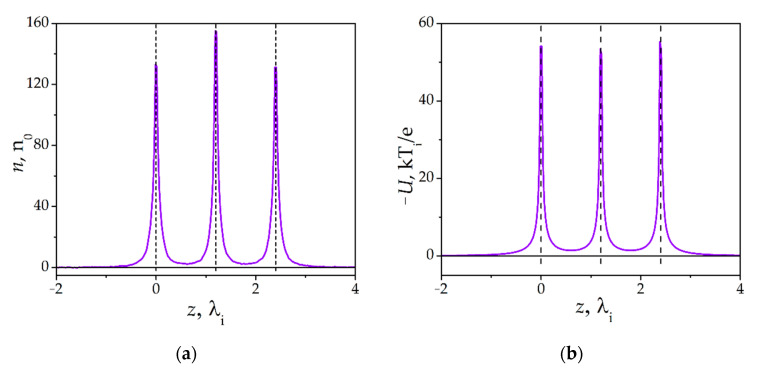
Self-consistent spatial distributions of the (**a**) space charge *n*(*ρ* = 0, *z*) and (**b**) plasma potential *U*(*ρ* = 0, *z*). *F_ext_* = −0.2, *l_i_* = 5, *E* = 0.

Figure 2 shows a cross section along the *z*-axis of the self-consistent spatial density distributions of the (a) space charge *n*(*ρ* = 0, *z*) and (b) plasma potential *U*(*ρ* = 0, *z*) in the vicinity of the dust particle chain. As already mentioned, the space charge *n*(*ρ* = 0, *z*) is expressed in units of *n_0_*. Far from the dust particle chain, *n_e_*/*n*_0_ ≈ *n_i_*/*n*_0_ ≈ 1. In the vicinity of the dust particles, the ion density *n_i_* increases by several orders of magnitude; in the same time, the electron density *n_e_* decreases. Thus, near a dust particle, the space charge is mainly determined by the ion density distribution (*n*(*ρ*, *z*) ≈ *n_i_*(*ρ*, *z*)). In the absence of an external electric field (*E* = 0), the space charge *n*(*ρ* = 0, *z*) is symmetric with respect to the chain’s center, i.e., the second dust particle. The ion cloud around the central dust particle is denser than for the first and the last dust particles. Furthermore, the ion clouds of the edge particles are anisotropic towards the central dust particle. This indicates that, when the dust particles are located close enough to each other, the ions orbiting around the first and third dust particles are partially recaptured by the central dust particle. It should be noted that, in this model, the condition of electric quasineutrality is met within the computational domain.

The ion clouds screen the dust particles charges with a screening length of the order of magnitude identical to *λ_i_*. Calculations in references [38,39] showed that, with the mean free path *l_i_* = 5 *λ_i_*, the dust particle charge is almost completely (1000 times) screened at distances of 3–4 *λ_i_*. Figure 2b shows that there is no complete screening of charges between the dust particles. As a result, there is a distortion in the ion density spatial distribution towards the central dust particle. Due to incomplete screening of dust particles charges, there will be Coulomb repulsion between them.

### 2.1. Influence of the External Confining Force

The self-consistent configuration of dust particles presented in Figure 1 and Figure 2 became possible only as a result of applying a confining external force *F_ext_* to the last (*k* = 3) dust particle. This force pushes dust particles closer to each other and prevents them from scattering. It is of interest to consider the influence of *F_ext_* on the self-consistent parameters of the dust particle chain, i.e., on the *Q_k_* and *Z_k_*.

**Figure 3:**
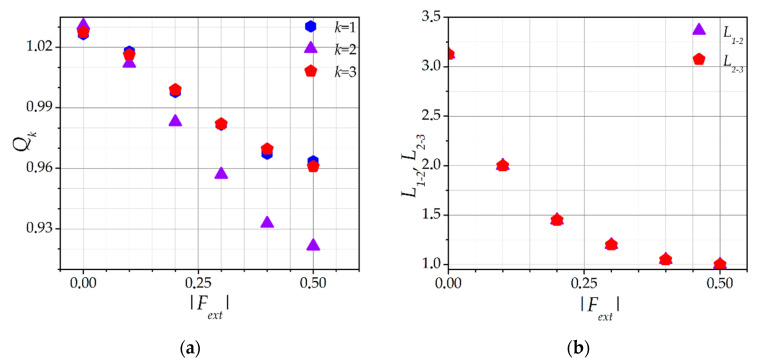
Dependence of the (**a**) self-consistent dust particle charges *Q_k_*, and (**b**) equilibrium interparticle distances *L_1-2_* and *L_2-3_* between the dust particles in the chain on the external force value *F_ext_*. *l_i_* = 5, *E* = 0.

Figure 3 shows the dependences of the (a) self-consistent dust particles charges *Q_k_* (k = 1, 2, 3) and (b) equilibrium interparticle distances *L_1-2_* and *L_2-3_* between the dust particles in the chain on the external confining force value *F_ext_*. These dependences were calculated for *l_i_* = 5 in the absence of an external electric field *E* = 0. Figure 3a shows that, with an increase of *F_ext_*, the dust particles charges *Q_k_* decrease. Furthermore, the charge of the central dust particle *Q_2_* decays faster. As the *F_ext_* increases, the particles approach each other, and their ion clouds partially merge. Ions orbiting around the first and third dust particles are frequently captured by the central dust particle. Figure 2 shows that, indeed, a denser ion cloud is formed around the central dust particle. In the denser ion cloud, the collisions of ions with neutral atoms are more frequent. Due to more frequent collisions, the ion flux towards the surface of the central dust particle increases. As a result (see Equation (5) in Section 3), the charge *Q*_2_ decreases.

Changes in the dust particles charges by themselves should not be associated only with the *F_ext_*. Thus, the dependence of the interparticle distances on *F_ext_* ought to be analyzed. Figure 3b shows the dependence of the distance between the first and second, *L_1-2_* = *Z_2_* − *Z_1_*, and the second and third, *L_2-3_* = *Z_3_* − *Z_2_*, dust particles on *F_ext_*. It is seen that the interparticle distances decrease with the increasing *F_ext_*. The confining force pushes the third dust particle into the chain, which, in turn, pushes the central dust particle with the Coulomb force *F_q_*. However, a decrease in the interparticle distances is also associated with a dust particle charge decrease (see Figure 3a), since the effect of Coulomb repulsion diminishes. Thus, Figure 3b explains and complements the dependence shown in Figure 3a. The phenomenon of a dust particle charge decrease with decreasing interparticle distance is observed experimentally [40,41].

It should be noted that, in the absence of an external electric field (*E* = 0), dust particles are equidistant from each other: *L* *= L_1-2_* = *L_2-3_*. In this case, the dust particle chain is stable without the presence of external confining force (*F_ext_* = 0). This is due to the fact that the forces acting on the dust particle from the ion cloud are either insignificant (for the first and third particles) or equal to zero (for the central particle) in the absence of an external electric field (*E* = 0). In the isotropic case, the ion cloud confines dust particles, and if random fluctuations from the equilibrium position occur, the forces acting from the ion cloud return the dust particle to its equilibrium position.

The equilibrium distance *L_max_* for the case *F_ext_* = 0 and *E* = 0 was also determined. It was approximately equal to *L_max_* ≈ 3.15 *λ_i_* (see Figure 3b), which, for the chosen set of parameters, was close to 300 microns and was in good agreement with the experimental data obtained under microgravity conditions on the basis of the PK-4 project [28].

**Figure 4:**
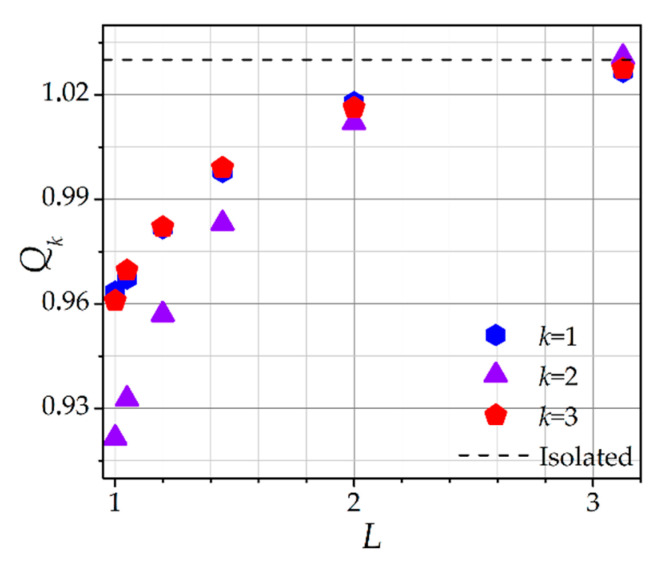
Dependence of the self-consistent dust particle charge *Q_k_* on the self-consistent interparticle distance *L*. *l_i_* = 5, *E* = 0.

The data presented in Figure 3a,b can be combined into a single curve, presenting the dependence of the dust particles charges *Q_k_* on the interparticle distance *L* (see Figure 4). It was seen that, with a decrease of the interparticle distance *L*, the dust particles charges *Q_k_* decreased. Moreover, for the central particle, the decrease of *Q_k_* occurred faster. The charges of the first and third dust particles were equal. For large *L*, *Q*_1_ = *Q*_2_ = *Q*_3_, which was due to the fact that the ions were no longer recaptured by the central dust particle.

### 2.2. Influence of the Ion Mean Free Path

**Figure 5:**
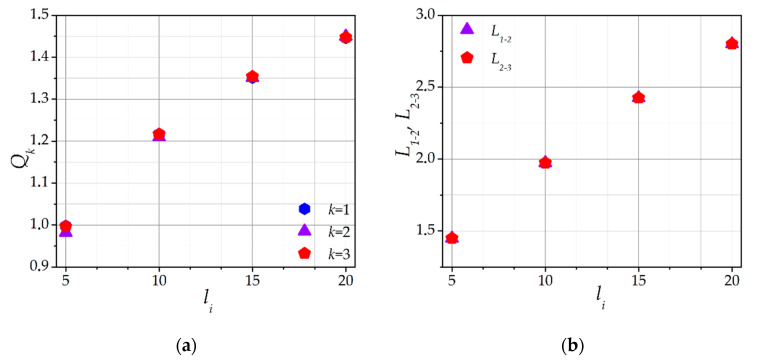
Dependence of the (**a**) self-consistent dust particle charges *Q_k_*, and (**b**) equilibrium interparticle distances *L_1-2_* and *L_2-3_* between the dust particles in a chain on the ion mean free path *l_i_*. *F_ext_* = −0.2, *E* =0.

The collisions frequency of ions with neutral atoms is one of the main parameters of this model, and the ion mean free path *l_i_* is determined in accordance with it. As shown in reference [38], *l_i_* is of great influence on the ion cloud formation and the dust particle charge; therefore, its effect on a dust particle chain ought to be considered. Figure 5a shows the dependence of the self-consistent dust particles charges *Q_k_* on the ion mean free path *l_i_*. This dependence was calculated for *F_ext_* = −0.2 and *E* = 0. It is seen that an increase of *l_i_* leads to an increase of *Q_k_*. This observation is consistent with the *Q*(*l_i_*) dependence calculated in reference [39] for isolated dust particles. Thus, for a dust particles chain, the dependence *Q*(*l_i_*) behaves qualitatively similarly as for isolated dust particles.

Figure 5b shows the dependence of *L= L_1-2_* = *L_2-3_* on *l_i_*. An increase of *l_i_* leads to a linear increase of *L*. Taking into account the increase of *Q_k_*, it can be stated that *L* increases due to the increase of the Coulomb repulsion forces. Furthermore, an increase *L* by itself also leads to an increase of *Q_k_* (see Figure 4). Thus, with a change of *F_ext_* or *l_i_*, a positive feedback effect is observed between the *Q_k_* and *L*. An increase in *l_i_* leads to an increase of the screening length and Coulomb forces; therefore, the chain is rearranged. This is consistent with the experimental results presented in reference [28], where it was shown that, at lower gas pressures, the interparticle distance increases. It is also worth noting that, for every chosen value of *l_i_*, the dust particles are equidistant for *E* = 0.

### 2.3. Influence of the External Electric Field

It was shown that, in the absence of an external electric field (*E* = 0), the parameters of the dust particle chain and surrounding plasma are distributed symmetrically with respect to the chain center. It was also demonstrated that, for *E* = 0, the presence of an external force *F_ext_* does not required for the confinement of dust particles in the chain. The presence of an external electric field distorts the ion cloud around an isolated dust particle [38,39]. Thus, the distributions symmetry around dust particle chain center will not be preserved. The effect of the external electric field *E* on the system of three dust particles will be considered further.

**Figure 6:**
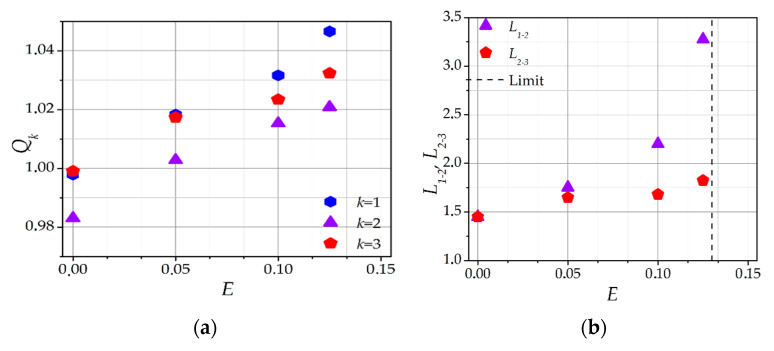
Dependence of the (**a**) self-consistent dust particle charges *Q_k_*, and (**b**) equilibrium interparticle distances *L_1-2_* and *L_2-3_* between the dust particles in a chain on the external electric field *E*. *F_ext_* = −0.2, *l_i_* = 5.

Under the influence of an external electric field, and as a result of periodic collisions with neutral atoms, ions generate a certain average drift velocity. Under the additional influence of the dust particle potential, ion focusing occurs, and a wake is formed downstream of the dust particle. As a result, a force begins to act on the dust particle, pushing it in the direction of the external electric field *E*. Thus, in the absence of an external confining force *F_ext_*, the dust particle chain becomes unstable, and the dust particles themselves scatter. To achieve a stable configuration of dust particles, the confining force *F_ext_* must be greater than the sum of the *F_q_* and *F_n_* acting on the last dust particle in the chain. The force of mutual repulsion *F_q_* is determined by the charges *Q_k_* and positions *Z_k_* of the dust particles. The dependences of these parameters on the external electric field strength *E* are shown in Figure 6a,b, respectively.

With an increase of *E*, *Q_k_* increases (see Figure 6a), which correlates with the data obtained for isolated dust particles [42]. Another feature of the electric field influence on a dust particle chain is that the symmetry in the dust particle charge distribution is violated: for *E* > 0, the charge of the first particle is always greater than the charge of the second and third particles, *Q*_1_ > *Q*_2_, *Q*_3_. This is due to the appearance of the ion focusing behind the first dust particle, as a result of which, ions fall much more frequently on the second and third dust particles.

The results presented in Figure 6 are in qualitative agreement with the results of the analytical models, numerical calculations, and experiments. In reference [30], it was shown that a chain of two dust particles placed in a plasma flow was stable if the charge value of a particle located downstream was less than the charge value of the first particle. In references [35,37], it was shown that, in a chain of three dust particles, the charge of the first dust particle was greater than the charge of the second dust particle, while the charge of the third dust particle was also greater than the charge of the second dust particle (*Q*_1_ > *Q*_3_ > *Q*_2_). The same effect was observed in the presented dependency (see Figure 6a). In reference [35], the changes in the dust particles charges were explained by the electrostatic lensing of ions on the dust particles. As a result of this lensing, the ion focusing effect appeared, and the ion flux to the second dust particle increased. According to the fluxes’ equality condition, this led to a second dust particle charge decrease. Electrostatic lensing was also performed by the second dust particle, which led to a decrease of the third dust particle charge. However, due to the fact that the charge value of the second dust particle was less than the charge value of the first dust particle, the effect of ion focusing was less. In reference [25], based on the experimentally determined positions of the dust particles, an identical effect was obtained by numerical simulation.

Based on the above, the dust particles charges should be greatly influenced by their location in space relative to each other. The dependence of their relative location to each other (interparticle distances) on the external field *E* is shown in Figure 6b. It can be seen that, with an increase of *E*, the distance between dust particles increases, which is associated both with an increase of the dust particles charges *Q_k_* (see Figure 6a) and with an increase of the forces acting on dust particles from the plasma space charge spatial distribution *F_n_*. The latter increases as a result of ion focusing. In contrast to the case *E* = 0, a warped ion cloud acts on a dust particle with a force pushing it in the direction of the wake. As mentioned before, in the absence of a confining force *F_ext_*, the dust particle chain is unstable. For the *F_ext_* = −0.2, the dust particle chain becomes unstable for an electric field strength greater than *E* > 0.13.

**Figure 7:**
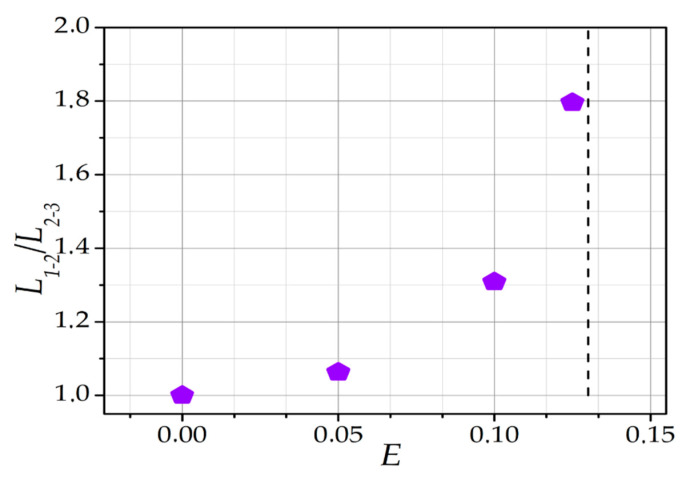
Dependence of the ratio *L_1-2_*/*L_2-3_* on the external electric field *E*. *F_ext_* = −0.2, *l_i_* = 5.

It should be noted that asymmetry arises in the interparticle distances under the influence of an external electric field, and *L_1-2_* becomes larger than *L_2-3_*. Additionally, it is interesting that the ratio *L_1-2_*/*L_2-3_* depends on *E* nonlinearly (see Figure 7). It can be observed that, with an increase of *E*, the ratio *L_1-2_*/*L_2-3_* increases nonlinearly, i.e., the middle particle is closer to the third particle than to the first (*L_1-2_* > *L_2-3_*). This is due to the fact that the force acting on the central dust particle from its ion cloud is significant. In the case of *E* = 0, the equidistance of the dust particles was a consequence of the *F_q_* = 0. The equilibrium condition then changed and, with it, the equilibrium interparticle distances.

The results presented in Figure 6b and Figure 7 are in qualitative agreement with the calculations of the equilibrium interparticle distances in the chain of the three particles performed in references [35,37]. In references [35,37], it was shown that the distance between the first and second dust particles was larger than between the second and third. This dependence also demonstrated a qualitative agreement with the experimental data presented in reference [25].

In this paper, smaller values of electric field strength and gas pressure (E < 0.13 V/cm, *p* < 10 Pa) were considered compared to those in PK-4 experiments (E~2 V/cm, *p* > 20 Pa) [28]. However, the results presented in Figure 7 can help to explain why, in DC mode, dust particles are not retained for a long time in the form of ordered string-like structures [28]. Under the conditions of the PK-4 experiment, the force that would hold the dust particle chains together is either absent or too small to confine dust particles. As shown in Figure 7, in this case, the dust particles will not form a stable dust chain. It should be noted that the presented version of the model does not take into account the AC electric field effects. The question of the nature of string formations and suppression in the AC and DC modes of PK-4 [28,43] will be addressed in the future.

## 3. Model

In this paper, an essential modification of the model described in references [38,39] is presented. In references [38,39], calculations of the plasma parameters were performed around an isolated dust particle under the action of an external electric field. The new model considers the process of self-consistent ordering of several dust particles into a one-dimensional chain. It takes into consideration the external forces, Coulomb repulsion force between dust particles, and self-consistent force defined by plasma potential spatial distribution around dust particles. The model can be conditionally divided into two main blocks: Block (1) calculation of spatial distributions of self-consistent plasma parameters (space charge and electric potential) and Block (2) calculation of self-consistent parameters of a dust particle chain (dust particle charges and distances between them).

The computational area is selected in the form of a parallelepiped in the Cartesian coordinate system, the sides lengths of which satisfy the condition *L_x_ = L_y_ << L_z_*. An external electric field *E_z_* (hereinafter referred to as *E*) acts in this region in the direction of the *z*-axis. On the *z*-axis (*x* = *y* = 0), *N* impenetrable spheres of radius *r_0_* are placed, i.e., representing the dust particles. The first particle *k* = 1 (*k* is the dust particle’s serial number in the chain) is always placed at the origin (*x* = *y* = *z* = 0). The remaining *N-1* particles are randomly placed on the z-axis, and their equilibrium positions are found using the procedure described further.

At the beginning of the calculations, a set of ions is generated uniformly in the computational domain. The initial velocities of ions are determined by the Maxwell distribution at room temperature (*T_i_* = 273 K), with an isotropic angular distribution. This velocity distribution corresponds to the appearance of a new ion after a charge exchange collision with a neutral atom. Ion trajectories are calculated using Newton’s motion equations, which include the potentials of charged dust particles, plasma space charge, and external electric field. The simulation of the motion of a single ion finalizes if it collides with either a dust particle (taken into account in the ion flux to the particle surface) or neutral atom. Then, a new ion is created.

As the first iteration, the ion trajectories are calculated in the potential spatial distribution, which is defined as a superposition of the Debye-Hückel potentials from *N* dust particles and the potential of the external electric field.

$$U_{0}(\rho ,z)=-\sum_{k=1}^{N}\frac{{\tilde{Q}}_{k}}{r_{k}}e^{-r_{k}}-\tilde{E}z,$$

(1) $$r_{k}{}^{2}=\rho^{2}+{\left(z-Z_{k}\right)}^{2},\;\rho^{2}=x^{2}+y^{2},$$

where *Z_k_* is the position of the *k*th dust particle on the *z-*axis.

The simplified form of Equation (1) is determined by the use of dimensionless variables. In the chosen representation, all variables denoting length are normalized to the ion Debye length $\;\lambda_{i}=\sqrt{kT_{i}/4\pi e^{2}n_{0}\;}$, where *n*_0_ is the plasma density. At the computational domain boundary, the plasma is considered unperturbed, and the densities of ions and electrons are normalized as *n*_0_ (*n_i_*(∞) ≈ *n_e_*(∞) ≈ *n*_0_). Spherical dust particles of the radius *r_0_* = 1 µm are considered, while the Debye ion length is set equal to *λ_i_ =* 100 µm (*r*_0_ = 1/100 *λ_i_*). All variables denoting energy are normalized to the ion thermal energy *k_B_T_i_*, and the variables denoting velocity and time are normalized to the values *V_Ti_ = √k_B_T_i_*/*m_i_* and *T* = *T* = *λ_i_√m_i_*/*k_B_T_i_*, respectively. In this representation, the dimensionless dust particle charge and the dimensionless external electric field are determined by the expressions:

(2) $$\tilde{Q}=\frac{e^{2}Z_{d}}{\lambda_{i}kT_{i}},\tilde{E}=\frac{eE\lambda_{i}}{kT_{i}}.$$

The considered problem of ion motion near a one-dimensional chain of dust particles under the action of an external electric field directed along this chain has a cylindrical symmetry. In this regard, the computational area is subdivided into segments according to cylindrical symmetry, and the time that each ion spent in each segment of space is accumulated. The spatial distribution of the ions residence time in the segments of the computational domain is directly proportional to the ion density spatial distribution *n_i_*(*ρ,z*), where *ρ* is the axis of the cylindrical coordinate system in the direction transverse to the *z* axis. The electron density spatial distribution *n_e_*(*ρ,z*) is determined by the Boltzmann distribution, taking into account the electric potential spatial distribution *U*(*ρ*,*z*). The space charge distribution *n*(*ρ*,*z*) is determined by the difference between the ions and electrons densities:

(3) $$n(\rho ,z)=n_{i}(\rho ,z)-n_{e}(\rho ,z)$$

The electric potential spatial distribution *U*(*ρ,z*) is determined by the Poisson equation:

(4) $$\nabla^{2}U(\rho ,z)=-n(\rho ,z)$$

This equation is solved using the finite difference method with the help of the Jacobi algorithm, where, in order to discretely represent Equation (4), a seven-point template for cylindrical coordinates is utilized [44].

The calculation of the plasma parameters around the dust particle chain is performed iteratively by Block 1. The algorithm of this calculation is the following:

1. At the initial stage, the electric potential spatial distribution is set to be equal to (1) (*U^t^*(*ρ*,*z*) = *U*_0_(*ρ*,*z*)).
2. Ions are generated, and their trajectories are calculated taking into account current *U^t^*(*ρ*,*z*).
3. According to the calculated ion trajectories, the ion density *n_i_*(*ρ,z*) is obtained. The electron density *n_e_*(*ρ,z*) and space charge *n*(*ρ*,*z*) are also calculated.
4. New iteration of the electric potential spatial distribution *U^t^*^+1^(*ρ*,*z*) is determined by Equation (4).
5. Step 2 is performed with substitution (*U^t^*^+1^(*ρ*,*z*)→*U^t^*(*ρ*,*z*)). The time step between iterations in Block 1 is selected in such a way that the electric potential spatial distribution would converge.

The main goal of this model is to determine the self-consistent parameters of a dust particles chain—namely, equilibrium (quasi-stationary) charges *Q_k_* and positions *Z_k_* of dust particles in the chain. These parameters are calculated by Block 2, described below. In Block 1, the dust particle chain parameters are considered to be constant.

The value of the *k*th dust particle charge *Q_k_* (the charges of different dust particles may differ) is determined iteratively, according to the ion *I_i,k_* and electron *I_e,k_* fluxes’ equality conditions:

(5) $$Q_{k}^{t+1}=Q_{k}^{t+1}-h(I_{i,k}-I_{e,k}).$$

The electron flux *I_e,k_* is determined in the same way as in [45], and the ion flux *I_i,k_* is obtained directly from the ion trajectories calculation. Here, *h* is a certain weighting factor specified for the numerical convergence of the dust particle charge *Q_k_* to a final value.

A more complicated issue is to determine the equilibrium positions *Z_k_* of dust particles in the chain. The position of the *k*th dust particle *Z_k_* is self-consistently determined by the balance of forces acting on it. In order to preserve cylindrical symmetry in the computational domain, only the forces acting on the dust particle along the *z*-axis are considered. Although in a real physical experiment there can be many such forces (including neutral drag, ion drag, thermophoresis, etc.), in this model, the only following forces are considered for simplicity:

- Coulomb repulsion of the *k*th dust particle from other dust particles, *F_q_*.
- The effect of the plasma space charge on the *k*th dust particle, *F_n_*.
- External force acting on the last dust particle, *F_ext_*.

In the classical experiment on dust particle crystals, the confining potential of the discharge chamber walls acts mainly on the boundary particles, which prevents dust particles from scattering under the action of repulsive Coulomb forces. In this model, *F_ext_* is set as a parameter. A superposition of forces acting on the *k*th dust particle in the chain follows:

(6) $$\begin{array}{l} F_{k}=F_{q,k}+F_{n,k}+F_{ext,k},\\ F_{q,k}=\sum_{j\neq k}{\tilde{Q}}_{k}{\tilde{Q}}_{j}\frac{Z_{k}-Z_{j}}{r^{3}},\\ F_{n,k}={\left.-\frac{\partial U(\rho ,z)}{\partial z}\right|}_{z=Z_{k},\rho =0},\\ F_{ext,k}=\left\{\begin{array}{l} F_{ext},k=N\\ 0,k\neq N \end{array}\right., \end{array}$$

where *F_n,k_* is the force of the space charge action on the *k*th dust particle, *F_q,k_* is the total Coulomb force acting on the *k*th dust particle, and *F_ext,k_* is the confining force acting on the last dust particle in the chain.

Due to the fact that the dust particle mass is several orders of magnitude greater than the ion mass, the calculation of the dust particles motion in the system has a critical effect on the calculation speed. To accelerate the calculations, a number of simplifications is utilized. One of the main simplifications is based on the fact that the dust particles positions are determined iteratively after each successful spatial potential distribution calculation performed by Block 1. In this case, the dust particles motion is described by the following formula:

(7) $$Z_{k}{}^{t+1}=Z_{k}{}^{t}+h_{z}\left(F_{q,k}+F_{{}_{\Delta n,k}}+F_{ext,k}\right),$$

where *h_z_* is a certain weighting factor used for iterative calculation of the dust particle positions.

It should be noted that one of the main disadvantages of the previous generation of models [38] is that its solution could only be static. This is due to the method fundamental feature, i.e., the process of accumulation of the ion residence time statistics is continuous, which meant that the space charge calculated at the early stages would affect the solution throughout the entire computation time and distort the final result. The main advantage of this approach is the possibility of using the mean field method, in which the ion motion is determined by its interaction with a potential calculated at the nodes of the computational mesh. However, due to the dust particles movement, such a preservation of statistics is illogical. Therefore, after the execution of Blocks 1 and 2, the following normalization is applied to the ion density spatial distribution:

(8) $$n_{i}{}^{t+1}(\rho ,z)=n_{i}{}^{t}(\rho ,z)\left(\frac{T_{cr}-\Delta t}{T_{cr}}\right),$$

where *T_cr_* is the computation time required in order to perform the process of normalization of the ion density spatial distribution, and Δ*t* is the computation time of the single iteration.

Thus, the calculation algorithm utilized in Block 2 is the following:

- The initial values for *Z_k_* and *Q_k_* are established.
- New values of the dust particles charges *Q_k_* in the chain are determined by Equation (5).
- New positions Z*_k_* of the dust particles are determined by Equation (7).
- The ion density is normalized according to Equation (8).
- Calculations in Block 1 are performed.

The execution of Blocks 1 and 2 is performed iteratively until all spatial distributions of the plasma parameters, *n*(*ρ,z*) and *U*(*ρ, z*), and the parameters of the dust particles chain, *Q_k_* and *Z_k_*, stop changing.

Despite the simplifications used in the model, it requires a significant computational capability to obtain the final self-consistent solution. The results shown in reference [38] were obtained by the model utilizing the parallel calculations implemented on the basis of the MPI architecture. The MPI made it possible to fully exploit the multithreading of a single central processing unit. However, even using the MPI for parallel computing, each self-consistent solution presented in reference [38] required two or more days to converge. In the case of a dynamic problem, where several moving dust particles are investigated, the performance drops sharply. It would take several hundred days to obtain single self-consistent solution if the MPI architecture was used. To increase the computational capability, a transition was made from MPI to CUDA architecture, where graphics processing units are utilized. With the use of graphic processors, the number of multithreads executed simultaneously increased a hundredfold, which made it possible to increase the model performance by ten times and reduce the computation time required to produce one self-consistent solution to one week. For numerical calculations, a video card with 1280 GPUs was used. A calculation of one separate regime for *N* = 3 particles took about 7 days. With an increase in the number of dust particles *N*, the calculation time required to determine the self-consistent parameters of the dust chain grows faster.

## 4. Conclusions

This paper presented a novel numerical model that allowed to determine the plasma parameters near a finite chain of dust particles. The main advantage of the presented method were its capability to automatically determine the self-consistent parameters of a one-dimensional chain of dust particles (charges of dust particles and interparticle distances) and self-consistent spatial distributions of the plasma parameters (space charge and potential) around it. As a product of numerical calculations, the following results were obtained, and the following conclusions were drawn:

- The spatial distributions of the space charge and plasma potential in the vicinity of a chain of three dust particles were calculated for the various parameters of the system. The symmetry of these distributions relative to the center of the dust particle chain in the absence of an external electric field was shown. It was also shown that the ion cloud density near the central dust particle was higher than that of the first and second dust particles.
- The dependence of the dust particles charges and interparticle distance on the external confining force, the ion mean free path, and the external field strength was obtained. It was shown how the configuration of a dust particle chain depended on those parameters.
- It was shown that a dust particle chain can be stable in the absence of external fields and forces and that dust particles will scatter at sufficiently strong external electric fields.
- It was shown that not only the spatial distribution of plasma around the chain of dust particles was distorted under the action of an external electric field, but asymmetry also arises in the parameters of the dust particle chain (in their charges and interparticle distances).

In this paper, calculations were performed for three dust particles that made it possible to study the behaviors of the fundamental parameters of the dust particle chains and the surrounding plasma. The presented results were in good agreement with the data obtained in other works experimentally, analytically, and numerically. At the next stage, it is planned to carry out calculations for a larger number of particles and in a wider range of parameters. The model proposed in this work can be further applied to describe the processes of dust particles ordering both in 1D chains, 2D monolayers, and 3D dust particle crystals.

## Data Availability

Data are available from the authors.
